# Hints for the Sick Room

**Published:** 1889-03-16

**Authors:** M. M. Basil

**Affiliations:** Author of "The Commoner Accidents to Life and Limb"


### Hints for the Sick-room.

By M. M. Basil, M.A., M.B., C.M., Author of "The Commoner Accidents to Life and Limb."

(*Continued from p.* 328.)

It is necessary to have a watch with a seconds hand for counting the pulse. The beats may be easily felt by placing the tips of the index and middle fingers of the right hand on the radial artery, just above and in front of the wrist. In cases where the artery is abnormal, a little search will find it to the right or left of its usual position, and even quite behind the wrist, or you may feel the artery of the other arm. You may count the number of beats: (1) For a whole minute (2) For half a minute, then multiply the number by two to obtain the beats for a minute. (3) For fifteen seconds, multiply it by four to obtain the number of beats for a minute. Here, however, any error made in your observation is increased fourfold. It is therefore preferable to divide the number of beats for fifteen seconds by three, to obtain the average for five seconds, and as there are twelve five seconds in sixty seconds, to multiply the quotient by twelve to obtain the beats for a minute.

It may be difficult to count the pulse in the usual way, owing to extremely quick and weak action of the heart. Unfortunately these are the cases where the observation must be repeated frequently, and this can only be done by the nurse. The beat of the heart may be counted here, either by feeling the heaving movement of the organ against the hand placed on it, or by hearing the heart-sounds directly or through a stethoscope. Each couple of sounds generally means only one beat of the organ. The quick succession of sounds is apt to confuse one, and hence it is best to count them for five seconds, two or three times, and then to reckon the average from the numbers so obtained.

The character of the pulse and the conclusion to which the chief varying conditions point are subjects which must be learnt by you, but cannot be considered here.

The respiration or breathing of the patient is another element which must be observed with care. Its number in the minute is best counted during sleep. Bodily movements or mental excitement affect it very easily. Hence, to obtain correct results, we must count it only after the patient has been in bed for some time, and the observation must be made without his knowledge. This is best accomplished by withdrawing the patient's attention by pretending to count the pulse, while the breathing is under observation. We may count the number of respirations by watching the movement of the chest or abdomen, by listening to the noise made by the inspiration or expiration of air, or by laying the hand which feels the pulse on the pit of the stomach and counting the heaving movement imparted to it. These methods are more likely to be successful than any gymnastic manœuvres or dancing about the bed to observe the patient from behind, as recommended in some books. Whatever the artifice used, the secret of it must not be betrayed to any patient, as that would at once destroy the utility of the measure.

The process of respiration may vary much in character. It may be thoracic or abdominal, quiet or noisy, slow or quick, deep, shallow, or jerky, painful or accompanied with pain. What is known as cerebral breathing is the most important variety for you to notice. Here the lips and check flap about with each respiration, the cheeks being blown out and the lips separated at each expiration. It is a kind of loud snoring with the lips, if such a description may be allowed. You may observe this variety of breathing when old people who have worn false teeth for years are deprived of such teeth and placed deeply under chloroform. Cerebral breathing is generally a symptom of very serious importance, and you should learn to recognise it so as to send for assistance at once. Should it be observed in an old patient who has had a fall, or is supposed to have been injured, no time must be lost in sending for help. Grave questions of life and death may depend on the use made of the few hours remaining to the patient.

The expectoration or sputum is worthy of careful observation. Its amount, consistence, colour, smell, and presence in it of blood in streaks or intimate mixture, are points which must be noticed. It should be remembered that some patients have a horror of letting it be known that they spit blood, and may therefore attempt to conceal the fact from you. Indeed, in all cases where unusual discharges or abscesses are expected to show their presence by evacuation, it is well to remember that from fear of being "cut or lanced" the patient may conceal facts from all about her. In more than one case of pelvic abscess the patient has succeeded in concealing repeated discharge of pus through the rectum, even when under careful observation.

The spittoon must be used regularly in cases of lung disease. It is best to choose one in which the contents are effectually covered. This prevents dust getting into the sputa, besides doing away with a sight which is certain to be unpleasant to everyone.

The nurse may materially help the patient in diminishing the restlessness from constant useless coughing by teaching him to how to cough effectually. To dislodge what causes irritation and consequent cough, it is necessary to take in a full breath previous to the expelling attempt made by the act of coughing. Sometimes hard masses, in the form of stones or casts, are coughed up after a violent paroxysm; these should be preserved for examination by the doctor.

The examination of the urine is one of the most fruitful sources of information in all diseases. In certain maladies, however, you may feel morally certain that a specimen of the urine is sure to be required for examination. You should therefore, in in all these conditions, take early measures to save the urine. Such are the first stages of all diseases where soreness of the throat is a symptom (scarlatina, suspected diphtheria—all sore throats), all cases of dropsy, of suspected poisoning, of fits of any description, of coma, drunkenness, delirium or severe headache, of suspected renal colic, of injury to the head and spine, of suspected pneumonia and cholera, of measles and purpura, and, fina ly, all conditions of high fever. The saving of urine in many of these cases is a matter of considerable importance, especially in male patients, because in the absence of such foresight the medical man must either remain in ignorance for a time on a very important point or he must pass the catheter. Now, catheterisation in the male patient is never quite free from danger, and where a little foresight on the part of the nurse will prevent the necessity of resorting to it, such foresight should not be omitted. The urine must be saved as a matter of routine as soon as these patients come under observation; but after the first visit of the medical man your duty in this respect must be guided by the instructions he leaves. There is no difficulty in saving the urine in every male patient seen outside a lunatic asylum, provided the necessary pains are taken. In children the urine should be collected immediately before evacuation of the bowels. In the most helpless patient it is perfectly easy to retain a V-shaped glass vessel in position between the thighs, by tying the limbs together at the ankles and above the knees. A soft pad should be placed between the limbs at both these points.

The reaction of the urine must be tested soon after its evacuation, and the amount passed in the twenty-four hours measured in ounces. Specimens kept for examination should be poured into clean urine glasses, and covered with paper to prevent the entrance of dust into the liquid. A label bearing the patient's name, ward, number, time of collection, and by whom collected, should be attached to the glass. In the female the urine must generally be drawn by the catheter if it is to be tested for albumen. When the contrary is the case, distinct mention of it should be made on the label. It is well to make sure that a patient who is inclined to mischief from idiocy and other causes gets no opportunity of putting into the urine articles which come from no human kidney.

There are some other particulars connected with the urinary functions which must be observed by the nurse: such are the frequency of micturition by day and by night; the colour and smell of the urine when passed compared with its characters after some hours; whether it is ropy or muddy when passed or only after some time; pain experienced before, during, or after voiding it. In a child, or comatose and dull patient, the expression of the face must be watched as an index of painful sensation. Continuous dribbling of urine must not be mistaken for passing water normally and reported as such. Incontinence of urine is most frequently a sign of retention of urine.

(*To be continued.*)

